# Extra-Abdominal Desmoid Tumours: A Review of the Literature

**DOI:** 10.1155/2012/578052

**Published:** 2012-08-16

**Authors:** A. P. Molloy, B. Hutchinson, G. C. O'Toole

**Affiliations:** Department of Orthopaedics, St. Vincent's University Hospital, Elm Park, Dublin 4, Ireland

## Abstract

Extra-abdominal desmoid lesions, otherwise known as aggressive fibromatosis, are slow-growing benign lesions which may be encountered in clinical practice. Recent controversies exist regarding their optimal treatment. Given their benign nature, is major debulking surgery justified, or is it worth administering chemotherapy for a disease process which unusually defies common teaching and responds to such medications? We present a literature review of this particular pathology discussing the aetiology, clinical presentation, and various current controversies in the treatment options.

## 1. Review

First described by McFarlane in 1832 [1], it was six years later that Muller [2] first coined the term desmoid, from the Greek word “desmos” meaning tendon-like. Also known as aggressive fibromatosis, desmoid lesions are benign locally aggressive slow-growing lesions that arise from deep musculoaponeurotic tissue.

Reports suggest that desmoid tumours account for 0.03% of tumours and approximately 3% of all soft tissue lesions [3]. Their incidence is approximately 3-4 cases per million population resulting in 900 cases annually in the United States [4]. Although case reports describe desmoid tumours in young children, the majority of cases occur between the ages of 15–60 years old, with a peak incidence 25–35 years [5]. There has been a well-documented preponderance in females with a 2 : 1 female/male ratio noted in the literature [5, 6].

## 2. Presentation

The majority of patients present with a painless swelling, however, should a lesion have adhered to deep structures, patients may present with contractures, pain, and dysfunction. When lesions expand to compress/invade local nerves, subsequent neurology may develop and result in radiculopathy, paraesthesia, or even motor weakness. 

The presence of a significant family history for abdominal or extra-abdominal lesions must be elicited. Gardner syndrome, first described by Gardner et al. in the 1950s [7], is an autosomal dominant condition characterised by the presence of polyposis of the colon, skull osteomas, papillary thyroid lesions, and sebaceous cysts. However it is also associated with an increased prevalence of extra-abdominal desmoid tumours, with a prevalence of approximately 15% in this cohort of the population [8].

Determining the presence of FAP in those presenting with desmoids lesions has become a clinical challenge, given the possibility of developing colonic cancer. A group from the Mayo Clinic reviewed their cohort of patients in the hope of finding clinical differences between sporadic and Gardner-associated desmoids lesions. There was a 16% incidence of FAP-associated desmoid lesions compared to the 84% of sporadic lesions. Although female distribution was higher in both groups, there was a more even ratio of cases in the FAP group. Although site and gender were not statistically significant, they did enable further statistical analysis through a Bayesian analysis to predict the probability of FAP-associated desmoid lesions [9].

A more recent Dutch study reviewing 519 patients put the incidence of FAP-associated desmoid lesions at 7.5%, lower than that of the Mayo Group. However, there was still an 800 fold increase for FAP patients of developing desmoid-type fibromatoses. Further analyses demonstrated an increased risk of FAP in patients presenting with desmoid lesions in the abdominal wall or intra-abdominally and in those under the age of 60. Given the higher risk in these subgroups, earlier intervention was recommended given the potential underlying malignancy [10].

Examination of a desmoid lesion typically demonstrates a smooth, mobile but firm swelling that is not readily transilluminable. As described, they can have deep attachments resulting in contractures, however, the overlying skin in usually unaffected with no erythema or tethering. Neurological examination must be performed to illicit any potential compressive symptoms.

## 3. Natural History

Although benign, with no metastatic potential, these lesions can progress to invade local neurovascular structures becoming symptomatic to the patient. In this instance, where surgery may be necessary, it is important to note the high recurrence rate of 15–77% [11], with a recent report from Dizdar et al. demonstrating a 52.6% recurrence rate at 8 years [12].

As with all lesions, wide resections with negative margins have resulted in lower recurrence rates. Various theories have been suggested to explain this high rate of recurrence including the fact that desmoid lesions can extend within muscle fibres themselves, making it difficult to achieve “clear margins” [12, 13]. As these are benign lesions, controversy exists regarding the amount of tissue that should be resected during surgery or whether surgery is needed in the first instance, given the potential debilitating sequelae to the patient [14]. The natural progression of desmoid tumours is erratic at best with some studies demonstrating 89% of cases progressing within the first 2 years of referral but not to greater than twice their size [14, 15].

Given its unpredictable nature, certain institutes have reported cases of spontaneous regression of these tumours in patients who would have required hindquarter amputation [16]. Although no correlation has been seen in these cases, the influence of hormonal changes has been postulated [17, 18].

## 4. Aetiology and Genetics

The majority of extra-abdominal desmoid tumours are idiopathic in nature. However, given their increased prevalence in those with Gardner syndrome [8], the roles of adenomatous polyposis coli (APC) gene mutations and *β*-catenin regulation have been investigated [19].

Although mutations in the APC gene are associated with Gardner-syndrome-related extra-abdominal desmoid lesions, sporadic lesions are a result of mutations in the *β*-catenin-coding CTNNB1 gene [20].

Catenins are proteins found within cadherin cell adhesion molecules. They play a central role in cadherin function by mediating the cadherin/actin filament network which in turn mediates their bonding ability [21]. *β*-Catenin, a member of the armadillo family of proteins, is believed to have an integral role in the development of desmoid lesions with elevated levels of this protein detected in such patients. Mutations of *β*-catenin can result in its stabilisation which in turn activates *β*-catenin-mediated T-cell factor/lymphoid enhancer factor-1-dependant transcription [22]. Animal studies, with such mutations, have demonstrated increased proliferation and invasiveness of increased number of fibroblasts [23].

The influence of *β*-catenin in regulating mesenchymal stem cell (MSC) differentiation is also believed to result in desmoid tumour formation. During fracture repair, elevated levels of *β*-catenin allow cells to maintain a fibroblast-like phenotype as opposed to an osteoblast-like phenotype [23, 24]. Thus, a gene mutation, present in MSCs, which codes for *β*-catenin stabilisation will allow cells to remain in an undifferentiated fibroblast-like state and continue on to form fibroblast-abundant desmoid lesions.

The CTNNB1 mutation (the gene encoding *β*-catenin) has a high incidence within extra-abdominal desmoid lesions compared to those associated with Gardener's syndrome. More recent studies have investigated the possible role of *β*-catenin mutation in recurrence rates among sporadic desmoid lesions [25, 26].

Recent analysis has shown an increase in matrix metalloproteinase-7 in patients with *β*-catenin mutations, another factor influencing the tumourigenicity of desmoid lesions. Similar studies investigated the correlation between VEGF and *β*-catenin, with a possible influence of Wnt/*β*-catenin pathway on angiogenesis. Interestingly, although one Japanese group demonstrated VEGF overexpression in conjunction with *β*-catenin disregulation, no significant difference was seen between recurrent and nonrecurrent groups. However, the role of angiogenesis in recurrence was confounded by the demonstration of increased microvessel density and high vascularity in the recurrent group, suggesting a distinct role of VEGF in its development [25].

At a genetic level, three specific mutations have been noted in those with CTNNB1 mutations, 2 at codon 45 and one at codon 41. Lev et al. from MD Anderson identified more than a threefold increased risk of recurrence in patients with a codon 45(F) mutation. Although negative margins were previously thought to be a major predictor of recurrence, an inherent genetic mutation may hold the key to predicting the natural progression of sporadic desmoid lesions [26].

The overexpression of COX-2 has been demonstrated in various cancers. However, with regard to desmoid lesions, this results in the overproduction of platelet-derived growth factor (PDGF) which acts as a mitogen for fibrocytes [27].

Previous surgery, trauma, and hormonal imbalance have all been suggested as possible risk factors for the development of extra-abdominal desmoid lesions. Given some response of desmoid lesions to selective oestrogen receptor modulators (SERMs) [28] and case reports of its development during pregnancy [29], the roles of hormone imbalance as a potential target for treatment have yet to be fully determined.

## 5. Radiological Investigation

Plain film radiology of the affected area is routinely normal. Occasionally, calcification may be seen or cortical erosion from the pressure effect of growing lesions adjacent to the bone. There is no extension into the medulla seen [30].

Ultrasonography is nonspecific, showing up as a poorly defined, hypoechoic soft tissue mass with larger lesions occasionally creating a posterior acoustic shadow [31].

**Figure 1:**
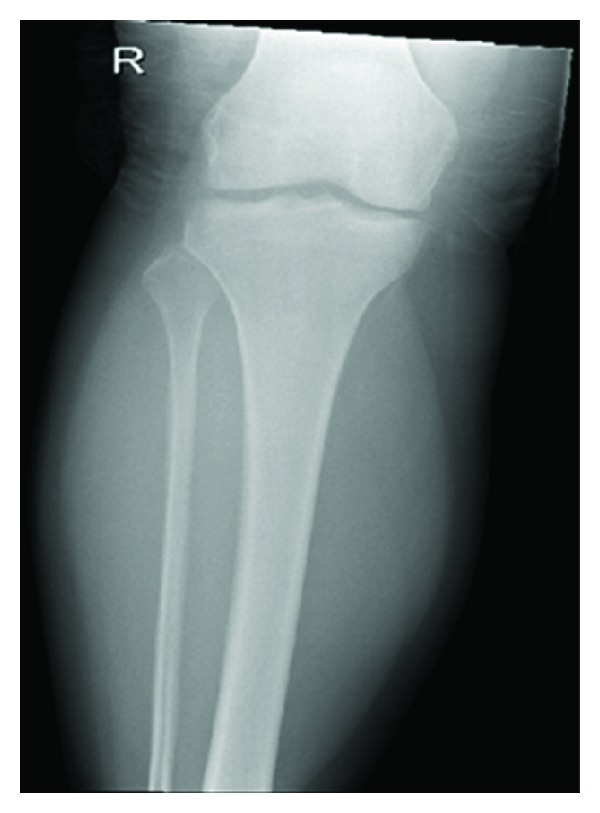
AP plain X-ray of right lower leg showing no abnormality.

**Figure 2:**
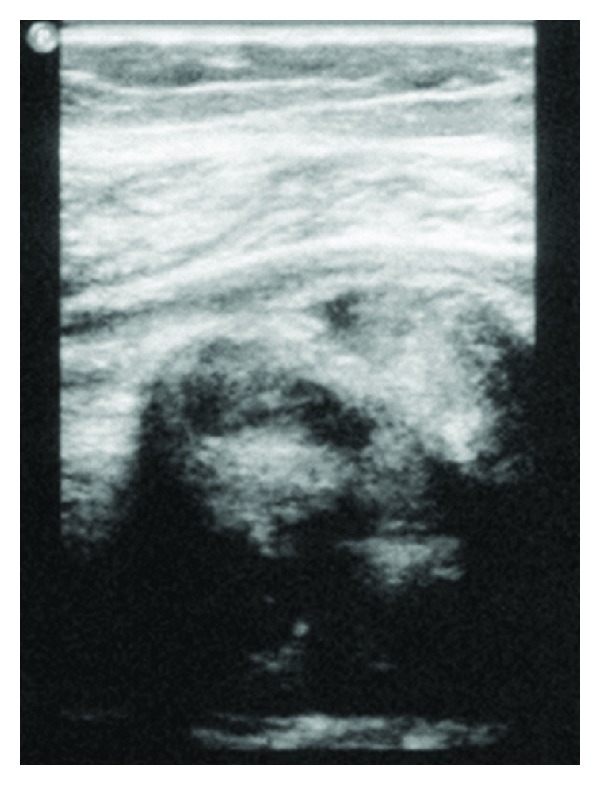
Ultrasound image of right lower leg showing a large heterogenous mass.

Computed tomography is also of limited value given the similar attenuation between muscle and extra-abdominal desmoid lesions. Following intravenous contrast administration, there may be increased enhancement due to the increased angiogenic activity within the lesion [31] (Figures 1 and 2).

**Figure 3:**
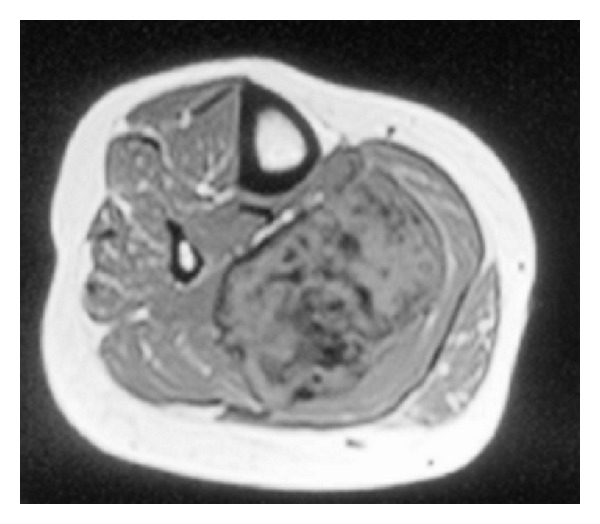
T1-weighted axial MRI of right lower leg showing heterogenous mass.

**Figure 4:**
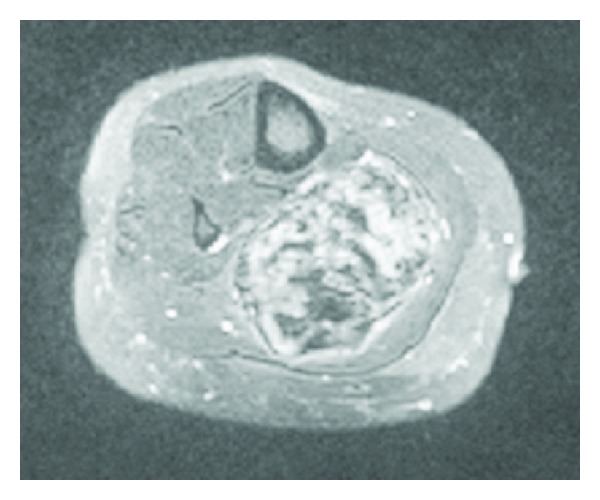
T2-weighted axial MRI of right lower leg showing heterogenous mass.

Magnetic resonance imaging (MRI) is the modality of choice to assess both the size of the mass and its intimate association with surrounding structures. Lesions on T1-weighted imaging are homogenously isointense with a high heterogenous signal on T2-weighted imaging. Intravenous contrast administration results in significant signal enhancement [32]. However, it is well documented that the degree of collagen within the lesion can influence the MRI findings, with an increased collagen component resulting in a decreased signal secondary to the hypocellularity [33]. MRI has the advantage of demonstrating any invasion into surrounding neurovascular structures compared to other radiological modalities (Figures 3 and 4).

## 6. Pathology

Gross examination of the specimen can reveal the lesion confined to the musculature or overlying aponeurosis. They vary in size and reveal a white, coarsely trabeculated surface when cut for cross-section analysis. In patients where the lesion has arisen in a postoperative site, it can be difficult to differentiate between the capsule and surrounding scar tissue [34].

**Figure 5:**
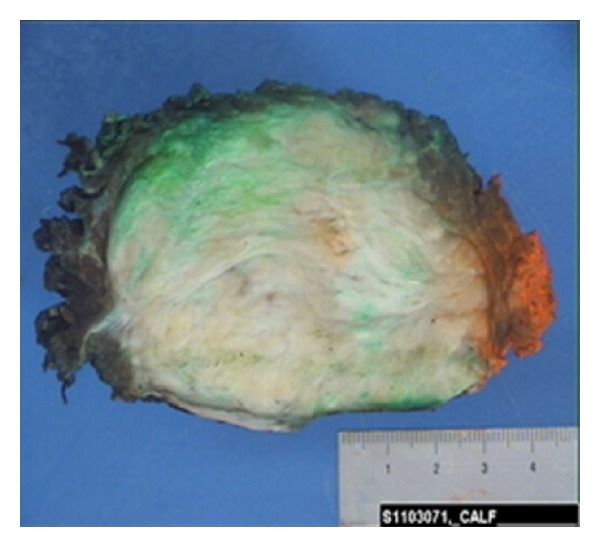
Cut surface of gross specimen (inked margins).

**Figure 6:**
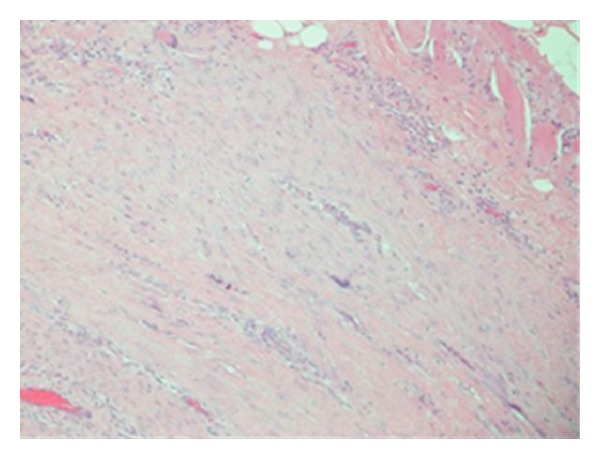
100x magnification showing spindle cells in fascicles with infiltration into muscle.

Microscopically, desmoid lesions are poorly circumscribed, with infiltration of surrounding soft tissue. High-power microscopy demonstrates the uniform appearance of elongated, spindle-shaped cells, lacking atypia. Collagen separates these cells with minimal cell-to-cell contact [34] (Figures 5 and 6).

Ultrastructurally, these lesions consist of elongated fibroblast-like cells. The cytoplasm have low mitochondrial numbers with prominence of both the Golgi apparatus and rough endoplasmic reticulum (which can be dilated and contain granular material). Stromal tissue is composed mainly of collagen and ground substance in variable amounts [35].

Immunochemistry analysis of desmoid lesions demonstrates a strong positivity for *β*-catenin, oestrogen receptor *β*, c-kit, and cathepsin D but negative for CD-24, oestrogen receptor-*α*, progesterone receptors and HER2 [15, 36]. Cytogenetic analysis reveals increased incidence of trisomies 8 and 20 with loss of 5q material noted in up 46% of cases [36].

## 7. Management

Current management of desmoid lesions is wide ranging but is linked by the need for a multidisciplinary approach. Given the unpredictable natural course of the lesion, treatment strategies can vary from observation to surgery and adjuvant therapy. Surgery, achieving clear margins, has long been the management option of choice. However, as these lesions are benign in nature, mutilating operations to achieve such goals have led to controversy among treating surgeons. With an increased knowledge of the cellular components of these lesions, more treatment options are being focused at various cellular receptors and at gene therapy to achieve a therapeutic response without the need for surgery. Radiotherapy and chemotherapy also play a potential role in the treatment of this disease.

## 8. Nonoperative

Desmoid tumours do not have a metastatic potential and therefore could be treated with a “wait and see” policy. This treatment option is advocated by Bonvolot et al. where in a retrospective analysis they demonstrated that 50% of patients benefited from front-line nonaggressive policy [37]. This strategy was applicable for both primary and recurrent cases. Given these figures, this group felt that it avoided the potential complications of both surgery and radiotherapy. It was also suggested that as surgery is the mainstay of treatment in most institutions, we could be over-treating half of these patients.

As with all oncological surgery, achieving negative margins is an important facet of decreasing risk of recurrence. However, in a benign lesion, which can be intermeshed within muscle fibres, it can result in extensive debulking surgery to achieve such a goal. In areas where complete resection may be difficult due to anatomic structures, a wait and see policy may be initiated. More recent studies have challenged the importance of negative margins given some positive outcome results following nonoperative management [38].

Extra-abdominal desmoid lesions create a challenge to the treating physician, as is evident from the array of treatment options available. A recent French study has aimed to differentiate the various subgroups with this pathology to ascertain the ideal management plan for each individual. Analysis of 436 patients indicated that age, tumour site, and size all influenced progression-free survival. Although macroscopic margins decreased overall outcome levels, there was no significant difference between negative margins and microscopic positive margins, further evidence to advocate a less invasive treatment protocol where possible [39].

## 9. Surgery

Wide margin surgical resection, despite its high rate of recurrence, has traditionally been the first-line treatment option [11, 40]. Surgery alone will render the patient disease-free, but this may be at the cost of permanent morbidity. It must be remembered that these are benign lesions and the treatment should reflect this, with a minimum of morbidity to the patient.

Huang et al. recently reported that on univariate analysis admission status (primary/recurrent), gender, tumour size, and margin status all correlated with local recurrence whilst size and margin status were independent prognostic factors on multivariate analysis. Local recurrence-free survival rates for primary disease at 5 years were 64% for those with positive margins compared to 92% at the same timepoint for those with negative margins [41].

The importance of clear margins is not a new concept with Rock et al. in 1984 demonstrating a greater recurrence rate in patients with an intralesional excision or with marginal excisions [42] which was later confirmed by a meta-analysis by Nuyttens et al. in 2000 [43].

Improved figures with the use of nonoperative modalities such as chemotherapy, radiation, NSAIDs, and hormonal treatments have now questioned the use of surgery as a front-line treatment option. Surgery now appears to be indicated in cases refractory to medical options. The use of amputation for this disease should only be used as a last attempt in recurrent patients with significant loss of function or chronic symptoms [44].

## 10. Radiation

The use of postoperative radiation as described by Nuyttens review of 22 series [43] demonstrated an increase in local control of the disease. In patients with positive margins the local control increased from 4% to 75% with the addition of adjuvant irradiation. There was also a positive result when used in patients with negative surgical margins.

These figures were more recently confirmed by Fontanesi et al.. Although only a small cohort of patients was included, they also demonstrated that through the use of postoperative irradiation (in the form of brachytherapy and/or external beam irradiation), patients with postoperative positive surgical margins demonstrated a 76% local control over a median follow-up period of 6 years. They advocated the use of total doses of greater than 50 Gy for microscopically positive groups and 56 Gy for gross residual disease [45].

As described earlier, there is a push towards conservative treatment as the first line of treatment for desmoid lesions. Rüdiger et al. recently reported similar recurrence rates in patients treated with radiation therapy alone to those treated with surgery and radiation treatment. However, there was some obvious selection bias in this series, as admitted by the authors themselves, with surgical candidates selected on a case-by-case manner. Even so, within the cohort for irradiation alone (external beam radiation therapy—median dose 50 Gy), follow-up MRI studies of these patients demonstrated a complete response in 3/15 patients, a partial response in 3/15, and stable disease in 8/15, with one patient suffering from disease progression [46].

There still appears to be a significant debate for the use of irradiation, whether alone or as an adjunct to surgery, in both primary and recurrent cases. The ideal dose required is between 50 and 60 Gy, with complications observed above this.

A recent long-term follow-up study investigating the benefit of radiotherapy for desmoid lesions has left more questions unanswered. A 15-year followup of younger patients (mean age 23.7 years at the time of radiotherapy) demonstrated overall survival and local regional control rates of 96% and 55%, respectively. Unfortunately over one-third of patients suffered significant complications during followup including pathological fractures, pain, and in-field skin malignancies. Given the incidence of complications, weighted against the overall benefit, the use of radiotherapy requires further investigation [47].

Nuyttens [43] paper references papers using older radiotherapy techniques, and although it does provide positive outcomes for the use of such modalities, care must be taken in using it as a basis for clinical practice. Like all treatment options, it should be used in a tailored approach for individual cases taking into account the possible long-term side effects.

## 11. Chemotherapy

Low grade lesions with no metastatic potential, such as desmoid tumours, should in theory not respond to chemotherapeutic agents given the low cell turn-over rates. With an increased knowledge of these lesions at a cellular level, responses have been demonstrated thus defying the traditional belief held at an oncological level.

Some of the initial responses to chemotherapeutic agents were described by Weiss and Lackman in 1989, which used a combination of vincristine and methotrexate on a weekly schedule with positive results [48]. Vincristine was later substituted for vinorelbine to decrease the incidence of neurological complications [49].

As mentioned, desmoid lesions contradict popular oncology teaching. Benign slow-growing lesions with no metastatic potential should not respond to chemotherapy. However as described, extra-abdominal desmoid lesions have shown positive response rates to various chemotherapeutic regimes. Controversy exists as to the correct combinations to use; given the benign nature of the lesion, it is imperative to prescribe the most effective treatment with the least side effect profile, notorious in chemotherapeutic drugs.

The French sarcoma group this year published positive results with approximately two-thirds of patients achieving disease stabilisation or objective response with combination of vincristine and methotrexate. However, anthracycline regimes demonstrated better objective responses but no difference in progression-free survival. The question of toxicity was raised in this study given the chemotherapy involved with the potential of pegylated liposomal doxorubicin restated given its lower side effect profile [50, 51].

More recently, doxorubicin-based treatment regimens have been used in the treatment of desmoid lesions. This has regularly been used in combination with dacarbazine with positive results [52] but with documented cardiotoxicity and myelosuppression. Gega et al. used a 96-hour continuous infusion of these two drugs followed by meloxicam with a complete response in three of seven patients and a partial response in the remaining four [53]. The majority of studies using such combinations have followup of over 5 years, thus confirming a long-lasting effect.

The use of pegylated liposomal doxorubicin has recently been reported from the Sarcoma Unit at the Royal Marsden Hospital, London [54], administered at a dose of between 40 and 50 mg/m² every four weeks for up to six weeks. Over 33% of patients treated demonstrated an objective response with the remainder deemed stable. There was no disease progression noted whilst on this regimen. Other positive outcomes included pain control and improved mobility.

These studies demonstrate an important role for cytotoxic agents in the treatment of desmoid lesions. Recent advances have resulted in an improved toxicity profile and positive outcomes, especially in unresectable, progressive lesions. However, noncytotoxic drugs such as NSAIDs also play an integral role in the management of the tumours.

In vivo studies with Cox-2 blockade have resulted in smaller desmoid tumours in a mouse model [55]. Although used in conjunction with doxorubicin and dacarbazine, Meloxicam (a cyclooxygenase-2 inhibitor) has had positive results when used alone in 22 patients with extra-abdominal desmoid lesions [56]. Following the exclusion of 2 patients in this study, 19 of the remaining patients 20 patients were evaluated as having stable disease or better (1 patient had a complete response with 7 having a partial response).

There has recently been increased interest in the potential role for tyrosine kinase inhibitors in the treatment of extra-abdominal desmoid tumours. The rapid response of patients with gastrointestinal stromal tumours to such antibodies is believed to be due, in large part, to the inhibition of c-kit RTK activity [57]. Imatinib mesylate, a selective tyrosine kinase inhibitor, has an antagonistic action towards PDGFR-*α* and PDGFR-*β*, along with c-kit, ABL, and ARG. The overall effect of blocking the receptor phosphorylation of these ligands is an inhibition of cellular proliferation and growth [54]. As described earlier, desmoid lesions demonstrate an increased production of PDGF, which may contribute to it becoming a potential target for treatment. Imatinib, a broader based tyrosine kinase inhibitor antagonises the same receptors along with vascular endothelial growth factor (VEGF) receptors [55]. Mace et al. and Skubitz et al. have recently published case series on the positive effect of both tyrosine kinase inhibitors with positive results [58, 59]. Mace et al., who described the use of imatinib mesylate, advocated further clinical research due to the increased c-kit and PDGR receptors in desmoid lesion. Heinrich et al., described the use of imatinib on 19 patients with variable results (3 patients had a partial response with another 4 having stable disease at one year). However, on immunohistochemical analysis of desmoid specimens, there was a higher rate of PDGFRB mutations compared to that of PDGFRA and KIT, along with significant mutation in the WNT pathway (84%—but of no clinical correlation with imatinib response) [60].

However, not all clinical studies have been entirely positive regarding the use of imatinib. The French sarcoma group demonstrated positive initial results (nonprogression rates at 3 and 6 month of 90 and 80%, resp.) but decreased at 12 months to 67%. The median time to progression was 25 months in this study [61]. These results were confirmed in the SARC trial with initial progression-free survival of 94% and 88% at 1- and 2-month follow-up appointments but decreased significantly to 66% at one year [62]. These results, although initially positive, demonstrated a limited role for imatinib alone but recommended it as part of a treatment armamentarium.

More positive results are being reported from the Memorial Sloan Kettering with the use of sorafenib. Sorafenib is a multikinase inhibitor with activity against Raf kinase and several receptor tyrosine kinases, including vascular endothelial growth factor receptor 2 (VEGFR2), platelet-derived growth factor receptor (PDGFR), FLT3, Ret, and c-kit [63].

The clinical benefit with the use of sorafenib was seen within 2 weeks in 70% of symptomatic patients. Longer radiological followup of a smaller cohort within the study demonstrated greater than 30% reduction in lesion size in 92% of patients. Encouragingly the majority of responders were in extra-abdominal lesions as opposed to intra-abdominal ones [64].

## 12. Cryoablation

With surgery resulting in damage to unaffected surrounding tissues, cryoablation has been advocated in the treatment of smaller extra-abdominal lesions. Kujak et al, reported positive results in their case series of 5 patients [65]. Alternating freeze-thaw exposure, with liquid argon administered through CT-guided probes resulted in successful relief of pain and a decrease in residual tumour by means of a minimally invasive technique. This may be of benefit in smaller lesions, however, is an unsuitable method of treatment in lesions with adjacent neurovascular structures.

## 13. Conclusion

Extra-abdominal desmoid lesions, although benign, can cause significant disability to patients when they encase surrounding nerves and vasculature. Surgery, the traditional management option, has always been associated with increased recurrence rates. Given the benign nature of the disease, there has been increased research into the nonoperative management options. These lesions are slow growing and by definition should not respond to chemotherapy. However, it appears to have numerous potential receptor targets that permit the use of both cytotoxic and non-cytotoxic chemotherapeutic agents, with positive results. The strategy for treatment is changing towards initial medical therapy, with surgery used in refractory cases. To illicit the benefit of chemotherapy and radiation in the treatment of these lesions, larger clinical trials based on Level 1 evidence are required, as the current literature is bereft of such studies.
